# Surveillance of Zoonotic Parasites in Animals Involved in Animal-Assisted Interventions (AAIs)

**DOI:** 10.3390/ijerph17217914

**Published:** 2020-10-28

**Authors:** Giulia Simonato, Patrizia Danesi, Antonio Frangipane di Regalbono, Giorgia Dotto, Cinzia Tessarin, Mario Pietrobelli, Daniela Pasotto

**Affiliations:** 1 Department of Animal Medicine Production and Health, University of Padova, viale dell’Università 16, Legnaro, 35020 Padova, Italy; antonio.frangipane@unipd.it (A.F.d.R.); giorgia.dotto@unipd.it (G.D.); cinzia.tessarin@unipd.it (C.T.); mario.pietrobelli@unipd.it (M.P.); daniela.pasotto@unipd.it (D.P.); 2 Istituto Zooprofilattico Sperimentale delle Venezie, viale dell’Università 10, Legnaro, 35020 Padova, Italy; pdanesi@izsvenezie.it

**Keywords:** animal-assisted interventions, pet therapy, parasites, dermatophytes, zoonoses, pet, settings, public health, Italy

## Abstract

Animal-assisted interventions (AAIs) are based on the establishment of a therapeutic relationship between animals and beneficiaries that is certain to provide positive effects, while currently, it reads as if AAIs aim at exposing stakeholders to potential risk of infection. The surveillance of zoonotic pathogens is necessary for guaranteeing common health. This study investigated the presence of potentially zoonotic parasites, including dermatophytes, in animals involved in AAIs. Between 2015 and 2017, 190 animals (equids, dogs, cats, birds, rabbits, rodents, and goats) were investigated. Anamnestic and management data were recorded. Individual faecal samples were analysed using a copromicroscopic procedure. Fur and skin were examined for ectoparasites during clinical examinations, and samples for mycological investigation were collected by brushing. Parasites were described in 60 (31.6%) investigated animals. Thirteen out of the 60 (21.7%) animals harboured potentially zoonotic parasites, mainly recovered in dogs (Ancylostomatidae, *Eucoleus aerophilus*, *Toxocara canis*, and *Giardia duodenalis*) and a cat (*G. duodenalis*). *Nannizzia gypsea* and *Paraphyton mirabile*, potential agents of cutaneous mycosis, were isolated in a dog and a horse, respectively. No ectoparasites were found. AAIs might represent a source of infections either directly or via environmental contamination. Thus, active surveillance is necessary and animal screenings should be planned and scheduled according to the risk of exposure.

## 1. Introduction

The positive impact of pets on the health of owners and people involved in animal-assisted interventions (AAIs) has been extensively documented [1,2]. These animals improve people’s perceptions of situations and encourage them to lead a better lifestyle due to daily exercise and the reduction in psychophysical distress [3,4]. Moreover, the guided interaction between people and trained animals helps patients to face health problems with a positive mood and to improve social awareness and communication in people with mental disorders [2,5,6,7].

The Italian national guidelines (2015) for AAIs of the Italian Ministry of Health describe three different typologies of interventions, i.e., animal-assisted activity (AAA), animal-assisted education (AAE), and animal-assisted therapy (AAT), that include professionals with growing expertise and whereby animals are coming into increasingly close contact with people/patients [8]. To date, these guidelines have provided some general criteria for ensuring that AAIs are correctly and evenly applied in the national territory; in particular, veterinarians are considered responsible for animal health, but no structured protocols to check the health and the potential parasitic zoonoses of animals operating in AAIs have been provided until now. In 2019, the National Health Institute summarised some considerations collected throughout the national territory during these four years of guideline application and elaborated some generic protocols for the control of health and wellbeing of the involved animals. This report represents only the starting point for improvement of the national guidelines currently in use toward improving the therapeutic efficacy of AAIs and facilitating more conscious management of the recruited animals [9].

Previous studies have already shown that apparently healthy animals involved in AAIs have a potential epidemiological role in asymptomatically carrying and even transmitting zoonotic pathogens to people [2,10,11]; this is of particular concern in AAT because the animal teams visit healthcare settings and interact with patients that could be immunocompromised for physiological and/or pathological reasons (e.g., age, pregnancy status, pre-existing pathologies, immunosuppressive therapy, and HIV/AIDS infection) [2].

This study describes preliminary data on the presence of potentially zoonotic parasites (i.e., endoparasites, ectoparasites, and dermatophytes) in various animal species working in AAIs in different settings (e.g., petting zoos, schools, and healthcare facilities); in particular, the first aim of the project was to evaluate if zoonotic parasites could circulate in clinically healthy animals and, if present, open a point of discussion in order to improve the Italian guidelines, currently in use. In addition, another aim is to verify the presence of non-zoonotic parasites in order to describe the animals’ health status. Finally, the last aim is evaluation of the real need to improve animal screening and to assess different protocols for checking the animals’ risk exposure to pathogens (e.g., habits, co-living with other animals, and lifestyle) with the overall purpose of guaranteeing both human and animal health.

## 2. Materials and Methods

Parasitoses in animals involved in AAIs were investigated over two consecutive years (July 2015–2017).

The survey was conducted on a voluntary basis, enrolling organizations from Northern and Central Italy.

Recruited animals were involved in AAIs conducted in different contexts and with increasing levels of exposure risk to pathogens, where interaction with animals was always present and mostly involved close contact. Since many animals attended more than one context, for each animal, the context with the major risk level of exposure was considered.

### 2.1. Description of Settings

The contexts in which the animals worked were divided into three main settings: health facilities, petting zoos, and schools.

The health facilities included hospitals, rest homes, care settings, and residential centres for physical and/or psychiatric disabilities where beneficiaries of AAIs—mainly AAT and AAA—being immunocompromised, with pre-existing pathologies, in immunosuppressive therapy, and stressful conditions and habits (i.e., patients with mental disorders that could have poor personal hygiene measures) might be exposed to the risk of potential zoonoses.

Petting zoos are a context in which pets and farm animals are placed together and live in the same environment wherein various activities with animals are offered (e.g., observational activities, projects on animal knowledge, school trips, and therapy for physical and mental disabilities, such as autism and relational problems); thus, they are open to a wide range of members of the public. In particular, animals belonging to petting zoos come into direct contact with a wide range of people, i.e., adults, teenagers, school-aged children, and toddlers. In this context, the risk represented by the age of children is that they may not yet have a competent immune system and/or appropriate hygienic habits.

Schools represent the last context in which animals interact only with toddlers and school-aged children (i.e., pre- and primary schools) who could be healthy and able-bodied and, in some cases, could have minor relational problems. The activities include school trips and inclusion projects with the aim of harmony and cohesion between beneficiaries and projects regarding the correct approach toward animals (AAA and AAE). In this latter context, as described for petting zoos, the risk of exposure is related to the not-yet-competent immune system and inappropriate hygienic behaviour of users.

During the sampling period, some animals were in training and were not yet involved in AAIs but they were examined and included in the study regardless due to their close relationship with their owner/trainer and cohabitation with other assisted therapy dogs. They belonged to a separated group referred to as “in training”.

### 2.2. Anamnestic Data and Physical Examinations

Firstly, anamnestic data on recruited animals were collected (i.e., species, sex, age, breed, antiparasitic treatments, context of AAIs, and close human–animal contact). Secondly, animals were physically examined to directly isolate ectoparasites (i.e., fleas, ticks, and lice) and/or to evaluate clinical signs related to them and to dermatophytosis (e.g., alopecia, scratching lesions, dermatitis, etc.) and/or to endoparasites (e.g., weight loss, pale mucosae, swollen abdomen, diarrhoea, vomitus, cough, etc.).

### 2.3. Faecal Sampling and Analysis

Animals involved in the project were sampled once. Faecal samples were individually collected, identified, and labelled. Faeces were conserved under refrigerated conditions and analysed within 48 h. Each stool sample (2 g for small animals and 5 g for farm animals) was submitted to a copromicroscopic technique consisting of sedimentation followed by a floatation step with sodium nitrate solution (specific gravity 1.3), as reported in the MAFF (Ministry of Agriculture, Fisheries and Food) manual (1986) [12]. Each faecal floatation was observed on a slide under a light microscope for the morphometric evaluation of helminths eggs and protozoa (oo)cysts at 100× and 400× magnification, respectively, according to existing keys [13,14].

### 2.4. Fur and Skin Materials Collection and Analyses

Fur and skin materials were individually collected using the Mackenzie brush technique, conserved at room temperature into a single clean plastic bag, and sent to the laboratory for mycological investigations. Brushes (and skin specimens) were cultured onto mycobiotic agar and incubated at 25 °C for at least 10 days. Cultures were checked daily and were considered positive when a single colony was morphologically suggestive of dermatophyte under the microscope as assessed by lactophenol cotton blue preparations. All dermatophyte colonies were isolated on Sabouraud dextrose agar for DNA extraction. Molecular investigations, including PCR targeting the ITS1/2 rDNA region [15] and sequencing of the amplicons, were performed in order to confirm fungal identity and to correctly assign taxonomy.

## 3. Results

A total of 190 animals (i.e., 94 equids, 56 dogs, 20 cats, 10 birds, 7 rabbits, 2 rodents, and 1 goat) were recruited.

**Figure 1:**
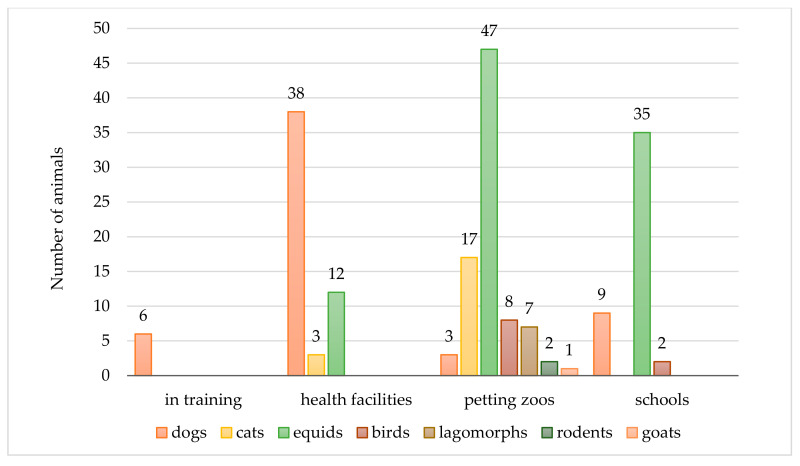
Number of study animals and their distribution in the main settings of animal-assisted interventions (AAIs).

The animals provided their activities within health facilities (*n* = 53, 27.9%, e.g., hospital, rest homes, and mental healthcare facilities), primary schools (*n* = 46, 24.2%), and petting zoos (*n* = 85, 44.7%). Six (3.2%) dogs were in training for AAIs. The prevalent species involved in AAIs were equids and dogs. In particular, dogs (*n* = 38/56, 67.9%) mostly attended health facilities, whereas equids worked mostly in petting zoos (*n* = 47/94, 50.0%) and with children/toddlers during school trips (*n* = 35/94, 37.2%). The animal species’ distribution among the different AAIs is shown in Figure 1.

### 3.1. Physical Examinations

At the physical examinations, all animals presented a good health status with neither parasitic arthropods (i.e., fleas, lice, and ticks) nor clinical signs referring to ecto- and/or endoparasites detected.

Most of the pet owners declared that their animals were regularly treated against ectoparasites (dogs with pyretroids or fipronil and cats with imidacloprid or fipronil) and against endoparasites with wide-spectrum anthelmintics (i.e., associations with avermectins for both dogs and cats) but no time frames for administration were reported to evaluate the effective regularity; the other animal species received treatments randomly or only in the case of clinical signs.

### 3.2. Zoonotic Parasites

Copromicroscopic analyses revealed the presence of at least one endoparasite species in 60/190 (31.6%) animals. In particular, 13/60 (21.7%) animals harboured potentially zoonotic parasites, mainly recovered in dogs (*n* = 12/13) as well as in a cat. In Table 1, the prevalence values of all parasites are reported.

The most prevalent zoonotic parasites were the respiratory nematode *Eucoleus aerophilus* and the enteric protozoa *Giardia duodenalis* (both, *n* = 4/17 positive dogs, 23.5%), followed by intestinal hookworms (i.e., Ancylostomatidae) and ascarids (i.e., *Toxocara canis*) (both, *n* = 2/17, 11.8%). The positive cat was infected with *G. duodenalis* (Table 1).

**Figure 2:**
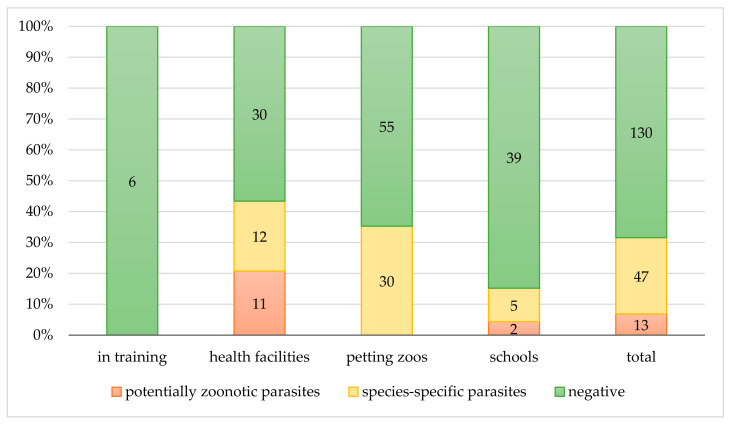
Copromicroscopic results. Distribution of negative and positive (zoonotic and species-specific) results among the contexts of the AAIs.

**Figure 3:**
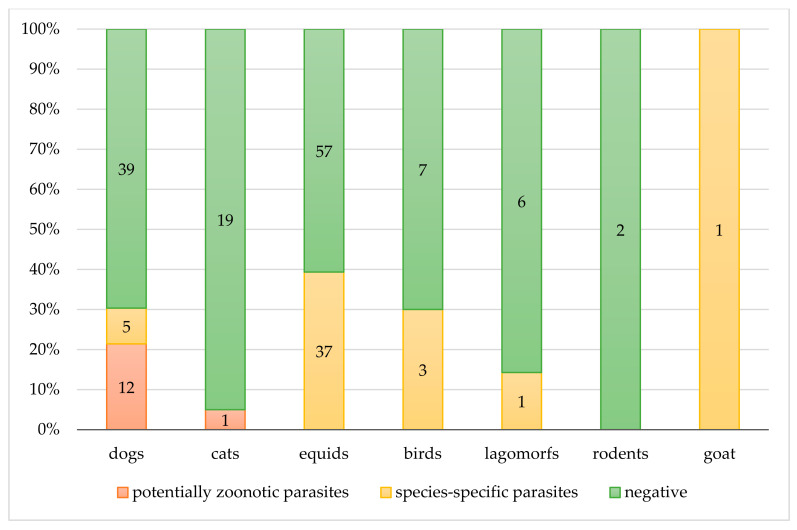
Copromicroscopic results. Distribution of negative and positive (zoonotic or species-specific) results in each animal species.

In addition, 10 dogs worked in health facilities and the other two in schools with children; the positive cat worked in rehabilitation and occupational therapy (i.e., health facilities) and also in schools among children (Figure 2 and Figure 3).

No endoparasites with zoonotic potential were recovered in the other investigated animals (Figure 3).

Among the studied contexts, the animals found to be affected by potentially zoonotic parasites attended health facilities and schools (Figure 2). In particular, 43.4% (*n* = 23/53) of animals attending health facilities were positive for at least one parasite species, and half of those, 11/23, presented zoonotic parasites, such as ancylostomatids, *E. aerophilus*, and *G. duodenalis*. In schools, 7/46 (15.2%) animals had parasites and 2/7 (28.6%) were dogs shedding *T. canis* ova. The cat shedding *G. duodenalis* cysts was not considered in the descriptive analyses of school settings because it was included in the category at major risk of exposure (i.e., health facilities) as previously stated in the Materials and Methods section; on the other hand, it is important to highlight that it represented a source of infection also for children in schools.

### 3.3. Non-Zoonotic Parasites

A total of 47/60 (78.3%) positive animals presented species-specific parasitosis. In particular, equids, birds, and lagomorphs showed prevalence values of 39.4% (*n* = 37/94), 30% (*n* = 3/10), and 14.3% (*n* = 1/7), respectively, and the only investigated goat was positive (Table 1). Among pet animals, only dogs were positive for a species-specific parasite (i.e., *Trichuris vulpis,* Table 1). Among the settings, animals in petting zoos were infected only by species-specific parasites (*n* = 30/85, 35.6%) (Figure 2). The 43.4% (*n* = 23/53) of animals attending health facilities were positive for at least one parasite species, and half of them (12/23, 52.2%) had species-specific parasites, such as intestinal strongyles (i.e., donkeys) and *T. vulpis* (i.e., dogs). In schools, 7/46 (15.2%) animals were positive for parasites, and among them, five had species-specific parasites (i.e., horses with intestinal strongyles) (Figure 2 and Figure 3).

### 3.4. Dermatophytes

Cultures positive for dermatophytes were described in two animals (2/190, 1%). More precisely, *Nannizzia gypsea* (ex *Microsporum gypseum*) was isolated from a dog and *Paraphyton mirabile* (ex *Arthroderma mirabile*, *Microsporum mirabile*) was detected from a horse. The positive animals worked with children and elders in a rehabilitation and occupational therapy context (i.e., health facilities).

## 4. Discussion

Pet therapy originated in the 1960s as an innovative therapeutic activity with the purpose of improving the health of patients through methodologies involving animals. The effectiveness of AAIs is strongly dependent on the quality of the relationship that the beneficiary establishes with the animal, which is strictly linked to the health and wellbeing of the involved animal.

In Italy, only in recent decades were AAIs considered, and their recent distribution along the national territory is greatly increasing to the point that the Ministry of Health, in 2015, published national guidelines to define the operating standards to evenly and correctly apply these interventions across the country. Moreover, the guidelines were issued for guaranteeing the health and wellbeing of involved patients and animals. In particular, veterinarians with expertise in AAIs and responsible for the health of animals operating in AAIs had no scheduled protocols to follow, and all the controls they advised and applied were complete at their own discretion. To date, there is a lack of widely shared criteria for the assessment of the health status and the wellbeing of the animals operating in the AAIs. Recently, the National Health Institute reported the most recent scientific acquisitions and, with the help of some experts, elaborated some generic protocols useful for this purpose, which represents a starting point for improvement of the national guidelines for AAIs.

Previous studies have already demonstrated that apparently healthy animals involved in AAIs have a potential epidemiological role in asymptomatically carrying and even transmitting zoonotic pathogens to people [2,10,11]; thus, the surveillance of potential zoonotic pathogens is mandatory for the health of both humans and animals and scheduled protocols to monitor their presence are strongly advised.

In our survey, multiple species of animals involved in AAIs were examined and their individual faecal samples and skin materials were analysed to evaluate the presence of parasites and dermatophytes, with particular interest in those that are potentially zoonotic.

Upon physical examination, all animals were in good health and no ectoparasites were detected, nor were there any clinical signs of parasites and/or dermatophytes in terms of recovery, even though a considerable number of animals, except dogs and cats, were randomly treated by owners against helminths and, only when considered necessary, against ectoparasites without a planned schedule.

Therefore, due to their asymptomatic status, one-third (31.6%, *n* = 60/190) of recruited animals presented unexpected parasitic infections. The antiparasitic treatments adopted by owners/trainers were not scheduled, nor were they regular or specific, and they were often based on the owner’s initiative. Thus, they were useless in controlling parasitosis. Since the national guidelines give free initiative to veterinarians for the assessment of the animal health status, the control of external and internal parasitosis is often managed at the owner’s initiative, which, in some contexts, seems to be not sufficient for avoiding unexpected parasites.

Of the positive animals, 21.7% (*n* = 13/60), comprising dogs and a cat, were affected by potentially zoonotic parasites, and these results are in agreement with data reported in Gerardi et al., (2017), where dogs involved in AAIs were investigated for parasites by copromicroscopic procedure, with positive results for *G. duodenalis*, *E. aerophilus*, *T. canis*, and *A. caninum*. These endoparasites are recognised worldwide as having zoonotic potential [3,16,17,18,19]. For instance, *Giardia* is a protozoan parasite, immediately infective for many mammals, including humans, when shed by the host [20], and the other helminths have parasitic elements, i.e., eggs/larvae, that need time to be infective once excreted and whose zoonotic potential is strictly linked to environmental contamination [21]. In particular, the risk for humans to be infected by *G. duodenalis* is still discussed and depends on prevalence rates of the protozoa and on its circulating assemblages. In fact, among the eight assemblages reported in the literature, A and B are considered zoonotic [20], and since they are frequently found in household animals [22,23], such as the dogs recruited in this survey, their presence should be strongly considered in therapy animals. In this survey, four dogs and one cat shedding *Giardia* cysts without clinical signs attended health facilities (i.e., hospitals and residential communities for psychiatric patients) and were involved in AAIs. Since no molecular investigations were carried out to identify *Giardia* assemblages isolated in pets, in our point of view, these animals should be considered as infected by potential zoonotic genotypes and treated accordingly. Considering the zoonotic helminths isolated as *A. caninum*, *E. aerophilus*, and *T. canis*, since they need time to be infective, the risk of human infection is strictly linked to the contaminated environment [21,24]. Thus, personal and environmental hygiene are strongly recommended to avoid undesirable parasitosis. The presence of zoonotic helminths and protozoa, mostly in dogs, suggests that the regular treatments declared by owners are not sufficient to control the parasitosis and, overall, not effective against protozoa infections. Moreover, it is strongly recommended that copromicroscopic examination be carried out at least monthly in these animals according to the European Scientific Counsel Companion Animal Parasites (ESCCAP) guidelines, with specific diagnostic tests (e.g., Baermann test, rapid coproantigen test, and PCR) eventually added, considering that dogs belong to the category at major risk of infections [25,26]. Based on the results of this study, monthly treatment with repellents or parasiticides against arthropods [27] is, in any case, advised for prevention, even if animals do not present visible ectoparasites or clinical signs referring to their presence.

Most positive animals (78.3%, *n* = 47/60) presented species-specific parasitosis, highlighting the need to improve the management of the animals with the purpose of guaranteeing their good health status and overall wellbeing—e.g., regarding the hygiene of animals, daily cleaning of shelters/stalls, frequent faeces removal, and routine copromicroscopic examinations followed by appropriate treatment. In fact, the style and living conditions of animals can influence their stress levels, which increase, for example, when the density of individuals in a restricted area grows, debilitating their immune system and making them more susceptible to infections, especially in poor management situations where the high environmental contaminations increase the risk of infection and pathogen transmission among animals [28,29,30,31].

Both *Nannizzia* and *Paraphyton* represent new genera included in the most recent taxonomy of dermatophytes [32]. Accordingly, “geophilic” dermatophytes have been renamed and allocated into separate genera, whereas the “zoophilic” *Microsporum* and *Trichophyton* species have maintained their classical name. The scientific community’s message is to maintain the classical nomenclature for fungal strains that are clinically relevant (including zoophilic and anthropophilic strains) and to address the “environmental” origin of geophilic dermatophytes. This concept is very important in terms of epidemiological approach. In our study, both *Nannizzia gypsea* and *Paraphyton mirabile* represent fungi that live and reproduce mostly in the soil. By contrast, zoophilic dermatophytes live and reproduce mostly on animals, and possibly on humans, if infected, and thus represent the real zoonotic risk. Results from this study indicate that the dog and horse were infected through contact with a contaminated environment, suggesting that patients, keepers, and other people operating in the same area are also exposed to the risk of infection by contact with the same soil. These results highlight the importance of going into deep identification at the species level for dermatophytes, even with molecular tools, in order to apply effective prophylaxis measures. Indeed, in our study, the infections mostly resulted from environment–host contact rather than animal–human contact. Geophilic species, as well as zoophilic species, may occur asymptomatically in mammals [33]. Thus, it is important that other animals also be monitored for dermatophytes in order to avoid their transmission to humans and other animals, which is favoured by their superficial localization on animal skin (zoophilic species) and the contamination of the environment by spreading of infective spores. In addition, animals should also be screened for dermatophytosis (fungal culture or, eventually, PCR) and excluded from activities until the results of cultures or PCR are confirmed to be negative. Then, it is recommended that topical treatments be adopted as recommended by the ESCCAP GL2 (2019), such as rinses or shampoos containing antifungal molecules, i.e., enilconazole or miconazole, 24 h before the activity, treating them as exposed animals [34]. Sodium hypochlorite solution at a 1:10 dilution and enilconazole solution could be adopted for washable surfaces and objects (i.e., brushes, combs, rugs, and cages) to control environmental dermatophyte spores. In animal shelters, it is advised that environmental surfaces be sampled to verify that disinfection has been effective.

The results emphasise that it is vital to routinely check animals, especially those that could be asymptomatic, that work daily with people that could be immunocompromised (e.g., patients, children, and elders with pre-existing pathologies) [17,35,36] or have inappropriate behaviour that favours infectious diseases, such as toddlers and patients with mental disorders, who could have poor hygiene behaviour [37]. A detailed control program, including laboratory analyses specific for each animal group, should be planned according to their activity, with particular attention given to animals attending health facilities and schools that are in close contact with people at major risk of infection.

### Strengths and Limitations

This study represents one of the first surveys in the national territory evaluating the parasitic health status of animals operating in AAIs. To the best of our knowledge, this is the first time that a wide range of animal species has been evaluated within different settings where the pathogen exposure risk was various. The collected results are preliminary and summarily informative but sufficient to suggest that zoonotic endoparasites circulate more than expected, especially in pets that are, among the animal species involved in AAIs, more easily recruited for activities in health facilities. Luckily, the copromicroscopic analyses were sufficient to identify most of the parasite species, even if molecular investigations would greatly improve the results of the study by adding new points of discussion and have to be done; unfortunately, molecular investigations were applied only for species identification of dermatophytes at the expense of *G. duodenalis* assemblages and hookworm species. Even if collected data do not prove the effective risks for stakeholders’ health, they call attention to potential ones. To evaluate the effective risks, further investigations need to be carried out—for example, molecular analyses on the isolate’s DNA. Moreover, the investigation of ectoparasites was during the clinical examination and no standardised methods of brushing were adopted. No arthropods nor clinical signs were registered but these data are not sufficient to conclude that they were absent; thus, the regular application of ectoparasiticides is suggested.

## 5. Conclusions

This study is a preliminary survey on parasitoses in animals involved in AAIs and represents a starting point for further investigation. Since the benefits of AAIs for people are extensively documented and the activities involving animals are spreading throughout the entire national territory, the risk of exposure to pathogens for both humans and animals should be taken into account. The collected data highlight the need to define criteria and tools to assess the wellbeing and health of animals operating in AAIs, considering overall asymptomatic animals that can carry potential zoonotic pathogens. For this reason, different protocols should be planned based on the real risk of exposure of animals to pathogens in relation to the lifestyle, management, and type of AAIs in which they are involved toward guaranteeing animal and human health according to the increasingly important concept of “One Health”.

## Figures and Tables

**Table 1:** Copromicroscopic results: number of investigated and positive (%) animal species and description of the isolated parasites.

Animal Species	Positive Animals	Subgroups of Positive Animals	Description of Isolated Parasites	
Animals (Total Number)	Total Number (%)	Number (%)	Zoonotic Parasites	Non-Zoonotic Parasites
Equids (94)	37 (39.4)	37 (100.0)		Intestinal strongyles
Dogs (56)	17 (30.4)	4 (23.5)	*Eucoleus aerophilus*	-
		2 (11.8)	*Giardia duodenalis*	-
		2 (11.8)	*Giardia duodenalis*	*Trichuris vulpis*
		2 (11.8)	*Toxocara canis*	-
		2 (11.8)	Ancylostomatidae	-
		5 (29.4)		*Trichuris vulpis*
Cats (20)	1 (5.0)	1 (100.0)	*Giardia duodenalis*	-
Birds (10)	3 (30.0)	2 (66.7)	-	*Capillaria* spp., coccidia
		1 (33.3)	-	*Capillaria* spp., *Heterakis* spp., coccidia
Lagomorphs (7)	1 (14.3)	1 (100.0)	-	Coccidia
Rodents (2)	0 (0.0)	0 (0.0)	-	-
Goat (1)	1 (100.0)	1 (100.0)	-	Coccidia
**Total (190)**	**60 (31.6)**			

## References

[1] Friedmann E., Son H. (2009). The Human Companion Animal Bond: How Humans Benefit. Vet. Clin. Small Anim..

[2] Bert F., Gualano M.R., Camussi E., Pieve G., Voglino G., Siliquini R. (2016). Animal assisted intervention: A systematic review of benefits and risks. Eur. J. Integr. Med..

[3] Chomel B.B., Sun B. (2011). Zoonoses in bedroom. Emerg. Infect. Dis..

[4] Brooks H.L., Rushton K., Lovell K., Bee P., Walker L., Grant L., Rogers A. (2018). The power of support from companion animals for people living with mental health problems: A systematic review and narrative synthesis of the evidence. BMC Psychiatry.

[5] Moretti F., De Ronchi D., Bernabei V., Marchetti L., Ferrari B., Forlani C., Negretti F., Sacchetti C., Atti A.R. (2011). Pet therapy in elderly patients with mental illness. Psychogeriatrics.

[6] Machová K., Procházková R., Eretová P., Svobodová I., Kotík I. (2019). Effect of Animal-Assisted Therapy on Patients in the Department of Long-Term Care: A Pilot Study. Int. J. Environ. Res. Public Health.

[7] Wijker C., Leontjevas R., Spek A., Enders-Slegers M.J. (2020). Effects of Dog Assisted Therapy for Adults with Autism Spectrum Disorder: An Exploratory Randomized Controlled Trial. J. Autism Dev. Disord..

[8] (2015). Italian National Guidelines in Animal Assisted Interventions. http://www.salute.gov.it/imgs/C_17_opuscoliPoster_276_allegato.pdf.

[9] Rapporti ISTSAN 19/4. http://old.iss.it/binary/publ/cont/19_4_web.pdf.

[10] Murthy R., Bearman G., Brown S., Bryant K., Chinn R., Hewlett A., George B.G., Goldstein E.J.C., Holzmann-Pazgal G., Rupp M.E. (2015). Animals in Healthcare Facilities: Recommendations to Minimize Potential Risks. Infect. Control Hosp. Epidemiol..

[11] Gerardi F., Santaniello A., Del Prete L., Maurelli M.P., Menna L.F., Rinaldi L. (2018). Parasitic infections in dogs involved in animal-assisted interventions. Ital. J. Anim. Sci..

[12] MAFF (Ministry of Agriculture, Fisheries and Food) (1986). Manual of Veterinary Parasitological Laboratory Techniques.

[13] Sloss M.W., Kemp R.L., Zajac A.M. (1994). Veterinary Clinical Parasitology.

[14] Di Cesare A., Castagna G., Meloni S., Otranto D., Traversa D. (2012). Mixed trichuroid infestation in a dog from Italy. Parasites Vectors.

[15] Irinyi L., Serena C., Garcia-Hermoso D., Arabatzis M., Desnos-Ollivier M., Vu D., Cardinali G., Arthur I., Normand A.C., Giraldo A. (2015). International society of human and animal mycology (ISHAM)-ITS reference DNA barcoding database-the quality controlled standard tool for routine identification of human and animal pathogenic fungi. Med. Mycol..

[16] Rabinowitz P.M., Gordon Z., Odofin L. (2007). Pet-Related Infections. Am. Fam. Physician.

[17] Abarca V., López J., Peña A.D., Carlos J., López C.G. (2011). Pet ownership and health status of pets from immunocompromised children, with emphasis in zoonotic diseases. Rev. Chil. Infectiol..

[18] Traversa D., Di Cesare A., Conboy G. (2010). Canine and feline cardiopulmonary parasitic nematodes in Europe: Emerging and underestimated. Parasites Vectors.

[19] Traversa D., Di Cesare A., Simonato G., Cassini R., Merola C., Diakou A., Halos L., Beugnet F., Frangipane di Regalbono A. (2017). Zoonotic intestinal parasites and vector-borne pathogens in Italian shelter and kennel dogs. Comp. Immunol. Microbiol. Infect. Dis..

[20] Feng Y., Xiao L. (2011). Zoonotic potential and molecular epidemiology of Giardia species and giardiasis. Clin. Microbiol. Rev..

[21] Traversa D., Frangipane di Regalbono A., Di Cesare A., La Torre F., Drake J., Pietrobelli M. (2014). Environmental contamination by canine geohelminths. Parasites Vectors.

[22] Claerebout E., Casaert S., Dalemans A.C., De Wilde N., Levecke B., Vercruysse J., Geurden T. (2009). Giardia and other intestinal parasites in different dog populations in Northern Belgium. Vet. Parasitol..

[23] Uehlinger F.D., Greenwoodc S.J., McClurea J.T., Conboy G., O’Handley R., Barkema R.W. (2013). Zoonotic potential of Giardia duodenalis and Cryptosporidium spp. and prevalence of intestinal parasites in young dogs from different populations on Prince Edward Island, Canada. Vet. Parasitol..

[24] Deplazes P., van Knapen F., Schweiger A., Overgaauw P.A.M. (2011). Role of pet dogs and cats in the transmission of helminthic zoonoses in Europe, with a focus on echinococcosis and toxocarosis. Vet. Parasitol..

[25] ESCCAP (2017). GL1: Worm Control in Dogs and Cats.

[26] ESCCAP (2018). GL6: Control of Intestinal Protozoa in Dogs and Cats.

[27] ESCCAP (2018). GL3: Control of Ectoparasites in Dogs and Cats.

[28] Beerda B., Schilder M.B.H., van Hooff J.A.R.A.M., de Vries H.W. (1997). Manifestations of chronic and acute stress in dogs. Appl. Anim. Behav. Sci..

[29] Simonato G., Frangipane di Regalbono A., Cassini R., Traversa D., Beraldo P., Tessarin C., Pietrobelli M. (2015). Copromicroscopic and molecular investigations on intestinal parasites in kenneled dogs. Parasitol. Res..

[30] Leonhard S., Pfister K., Beelitz P., Wielinga C., Thompson R.C.A. (2007). The molecular characterisation of Giardia from dogs in Southern Germany. Vet. Parasitol..

[31] Ortuño A., Castellà J. (2011). Intestinal parasites in shelter dogs and risk factors associated with the facility and its management. Isr. J. Vet. Med..

[32] De Hoog G.S., Dukik K., Monod M., Packeu A., Stubbe D., Hendrickx M., Kupsch C., Stielow J.B., Freeke J., Göker M. (2017). Toward a Novel Multilocus Phylogenetic Taxonomy for the Dermatophytes. Mycopathologia.

[33] Choi J.S., Gräser Y., Walther G., Peano A., Symoens F., De Hoog S. (2012). Microsporum mirabile and its teleomorph Arthroderma mirabile, a new dermatophyte species in the M. cookei clade. Med. Mycol..

[34] ESCCAP (2019). GL2: Superficial Mycoses in Dogs and Cats.

[35] Khan M.A., Farrag N. (2000). Animal-assisted activity and infection control implications in a healthcare setting. J. Hosp. Infect..

[36] Mani I., Maguire J.H. (2009). Small animal zoonoses and immuncompromised pet owners. Top. Companion Anim. Med..

[37] MacPherson C.N.L. (2005). Human behaviour and the epidemiology of parasitic zoonoses. Int. J. Parasitol..

